# Contextual influences on health worker motivation in district hospitals in Kenya

**DOI:** 10.1186/1748-5908-4-43

**Published:** 2009-07-23

**Authors:** Patrick Mbindyo, Lucy Gilson, Duane Blaauw, Mike English

**Affiliations:** 1 Kenya Medical Research Institute Centre for Geographic Medical Research Coast-Wellcome Trust Collaborative Programme, P. O. Box 43640-00100 GPO, Nairobi, Kenya; 2 School of Public Health and Family Medicine, University of Cape Town, Observatory, 7925, South Africa; 3 Health Policy Unit, London School of Hygiene and Tropical Medicine, Keppel Street, London WC1E 7HT, UK; 4 Centre for Health Policy (CHP), School of Public Health, Faculty of Health Sciences, University of the Witwatersrand, P.O. Box 1038, Johannesburg, 2000, South Africa; 5 Department of Paediatrics, University of Oxford, John Radcliffe Hospital, Oxford. UK

## Abstract

**Background:**

Organizational factors are considered to be an important influence on health workers' uptake of interventions that improve their practices. These are additionally influenced by factors operating at individual and broader health system levels. We sought to explore contextual influences on worker motivation, a factor that may modify the effect of an intervention aimed at changing clinical practices in Kenyan hospitals.

**Methods:**

Franco LM, et al's (Health sector reform and public sector health worker motivation: a conceptual framework. Soc Sci Med. 2002, 54: 1255–66) model of motivational influences was used to frame the study Qualitative methods including individual in-depth interviews, small-group interviews and focus group discussions were used to gather data from 185 health workers during one-week visits to each of eight district hospitals. Data were collected prior to a planned intervention aiming to implement new practice guidelines and improve quality of care. Additionally, on-site observations of routine health worker behaviour in the study sites were used to inform analyses.

**Results:**

Study settings are likely to have important influences on worker motivation. Effective management at hospital level may create an enabling working environment modifying the impact of resource shortfalls. Supportive leadership may foster good working relationships between cadres, improve motivation through provision of local incentives and appropriately handle workers' expectations in terms of promotions, performance appraisal processes, and good communication. Such organisational attributes may counteract de-motivating factors at a national level, such as poor schemes of service, and enhance personally motivating factors such as the desire to maintain professional standards.

**Conclusion:**

Motivation is likely to influence powerfully any attempts to change or improve health worker and hospital practices. Some factors influencing motivation may themselves be influenced by the processes chosen to implement change.

## Background

A number of factors ranging from the individual to national level operate together to influence how health workers take up interventions to improve their work practices [1-5]. Often this influence works through the local personal, educational, professional, community, or institutional environment in which work takes place, or the social, cultural, economic, and political environments more generally [1,2]. Specific efforts within these environments to manage health worker actions include a broad set of incentives and sanctions [1]. At the individual health worker level, many of these influences are understood to affect a worker's motivation to act in desired ways. Thus, understanding those factors that influence worker motivation is important when trying to explain why interventions that rely on changing worker behaviour succeed or fail.

However, worker motivation and its influence on changing clinical practices of health workers in low-income settings [2,6,7] is rarely explored as a major factor that may mediate or modify the effects of interventions [2,7-9]. More usually, studies of health worker's motivation explore determinants of motivation by examining the subjective perceptions of health workers [8,10-15] either to understand effects of health sector reforms on worker performance [10,11,14], or influences of performance management on worker motivation [8,11,13].

We are conducting a study of an intervention aiming to improve the quality of care for children in Kenyan government hospitals. The study design and intended interventions have been described elsewhere [16,17]. Conscious of the fact that the characteristics of the hospitals as organisations, their health workers and their interaction with the research team might be major factors affecting implementation the research design also aimed to explore these issues [7]. One topic of focus was, therefore, hospital staff motivation. We reasoned that exploration of motivation even if only at baseline would provide us with an improved understanding of factors that might affect the intervention's eventual success.

We have described elsewhere our efforts to develop a quantitative measure of motivation to inform analyses of the outcomes of the intervention project [18]. Here we describe, based on an exploration of motivation, the results of qualitative investigations in the study hospitals that help describe the health system context within which the intervention was delivered. In accompanying work, we also describe the hospitals as contexts from a more traditional quality of care perspective [19], the process of intervention [20] and reported barriers to use of clinical practice guidelines [21]. These detailed descriptions will, we hope, provide a thick description of the hospitals we studied as 'typical' contexts providing health care services in rural areas of Kenya. In so doing, we aim to improve understanding of the broad range of issues affecting attempts to change hospital practices and help others critically evaluate the generalisability of our future reports on the effectiveness of the intervention.

## Methods

### Theoretical approach

**Figure 1:**
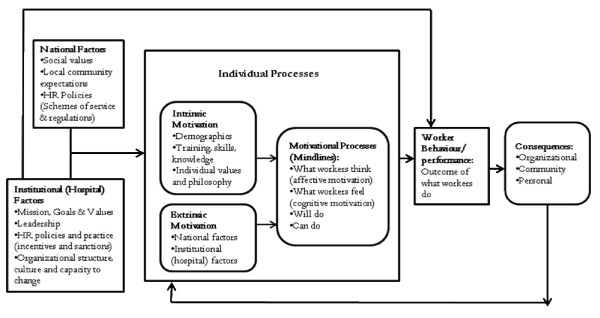
**Influences on worker motivation**.

The use of qualitative methodology was to explore the depth, richness, and complexity of staff motivation in district hospitals prior to the practice change intervention being implemented [22-24]. We have adapted Kanfer's [25] model that outlines the complex play of forces that influence motivation that operate at individual, organisational, and societal levels [9,25]. It divides determinants of motivation into 'will do' (*i.e*., adoption of organisational goals) and 'can do' components (*i.e*., mobilisation of personal resources to achieve joint goals) [25]. The adaptation of Kanfer's [25] model was informed by Franco *et al*.'s[7,9] work that extended the model to provide a clearer understanding of the various factors that affect workers motivation before designing interventions that explicitly or implicitly affect motivation (see Figure 1).

### Tool development

Based on these theoretical considerations, Key Informant Interview (KII) and Focus Group Discussion (FGD) tools were developed. The KII tools, in particular, were developed with regard to the cadre of likely respondents, (junior cadres, middle, and senior level management). Each guide had five sections comprising questions and probes with flexibility to explore issues affecting particular cadres, such as doctors or nurses. The qualitative guides were piloted in two, non-study public hospitals in Kenya to test for clarity of questions, health workers' comprehension of the tools, and to gain preliminary insight into respondents' perceptions of motivation. All tools were revised and finalised after this piloting.

### Sampling and data collection

The selection of study hospitals has been described in full elsewhere. Briefly, they comprised eight rural district hospitals from four of Kenya's provinces [16] selected to represent a range of institutional, geographic, socio-economic, and epidemiological settings. The nature and scope of the study was discussed with study hospitals prior to any data collection. Once they had agreed to take part, the first major contact with the research team was the conduct of two-week baseline surveys run in parallel across the country. These surveys focused on a broad quality of care assessment described fully in an accompanying manuscript [19]. The qualitative data described here were collected by the lead author during one-week visits to each hospital made after the departure of the baseline survey teams and before the results of baseline surveys were provided to the hospitals. These visits were conducted during August and September 2006 prior to any intervention. A convenience sampling approach was used to select participants to be interviewed in English (the language of all primary, secondary, and tertiary education in Kenya). Because the numbers of some key informants (hospital chief executive officer (CEO), administrator, matron, and ward in-charges) and clinicians (doctors and clinical officers, or COs) were few, an effort was made to interview all present during the one-week visit.

In this study, the main focus of data collection was professional staff working in areas with regular contact with sick children in their day-to-day work because the intervention was aimed at improving paediatric care. FGDs were conducted among nurses (especially in maternity and child health sections) because they form over 50% of the clinical staff in the hospitals. FGDs were mainly done in the late afternoons because workloads reduced considerably in this period. Throughout the one-week visits to hospitals the principal investigator (PI) was an engaged observer of health worker roles, attitudes, and practices, and the functioning of the hospital as an institution, keeping detailed field notes to supplement interview data.

### Data analysis

In response to some sensitivity about tape-recording, detailed notes of interviews and group discussions were the primary data record with tape recordings used to supplement these where possible (in fewer than 20% of interviews). All notes of interviews undertaken in the field were transcribed into MSWord 2003 (Microsoft Corporation, USA) by the PI. These were then imported into NVIVO7 software (QSR International Pty Ltd, Australia) categorised by type of interview (FGD, small group, key informant, or observations). Each transcript had a unique identifier comprising of date, hospital code, type of interview, and participant type, allowing exploration of data by subgroup (*e.g*., health worker cadre).

Coding into themes was carried out in a three-fold manner. The initial coding process followed the directed content analysis procedure [26] where theory was used to guide the coding process. This was done during fieldwork where the investigator examined his notes at the end of every day and identified any issues that needed further exploration or clarification. This was achieved by returning to the same individual or exploring arising issues with new participants. The second was during transcription where, independent of the first phase, prominent issues were marked for further exploration. Finally, after importing the transcripts to NVIVO7, conventional coding (where coding categories are directly derived from the text data [26]) was performed without reference to the results of the first two coding processes. Results from the three processes combined with views of a second, independent reading by a second investigator (ME) of more than half of the transcripts, and insights from on-site observations were reviewed and used to derive relevant major thematic categories. Codes that initially seemed to be different were re-examined and found to provide additional explanation for the larger categories, a process that improved our explanatory ability.

### Ethical issues

Ethical clearance for these studies was obtained from Kenya's National Ethics Review Committee, and permission was gained from the heads of each hospital before work started. Written consent was sought for interviews and FGDs from the study participants.

## Results

A total of 185 staff comprising of hospital directors, matrons and administrators (n = 19); nurses (n = 92), doctors (n = 13), pharmacists (n = 4) and COs (n = 36); and other paramedics comprising of laboratory, dental, orthopaedic, and pharmaceutical technologists (n for group = 21) contributed data (Table 1). Overall, the majority of respondents were female, which concurs with the findings of the 2004 MoH Human Resource Mapping exercise that found more female workers (52.7%) than male (47.3%) in Kenya's health workforce [27]. In Kenya, COs are a form of substitute physician undergoing a four-year academic and internship training. They are twice as numerous as doctors in the health system, being major providers of clinical services in rural hospitals. Their pay is comparable to that of nurses and usually less than 50% of that of even junior physicians.

**Table 1:** Numbers of interviews by hospital

*Hospital Code*	*Key Informant Interviews*	Focus Group Discussions*	*Small Group Interviews#*
H1	14	0	4 (3,2,3,2)
H2	15	1 (7)	3 (4,2,3)
H3	14	1 (5)	2 (2,2)
H4	6	1 (7)	2 (2,2)
H5	9	1 (10)	0
H6	7	0	4 (3,4,3,4)
H7	13	1 (10)	3 (2,3,4)
H8	12	0	2 (3,3)

**Total**	90	5 (39)	20 (56)

*To be classified as a focus group discussion, an interview had to have at least five members of staff excluding the interviewer. The brackets show the number of staff present in that particular session.

#Small group interviews comprised of discussions that had two to four staff members. The brackets show the number of staff present in the sessions held.

All FGDs (n = 5, with 39 participants) were carried out in the maternity and child health sections. In other areas, low staff numbers only made it possible to conduct individual (n = 90) or small group interviews (n = 20, with 56 participants). All respondents found the study and its topics to be very timely. Even so, a few respondents (about 5%) found questions relating to promotions, salary, and training to be very sensitive and seemed guarded when addressing these issues.

In line with our conceptual framework and our intention to provide a rich, contextual description of the hospitals studied, we present our data stratified by the level at which factors may operate to influence motivation (national, institutional, and personal). We then present a description of their effects in discussing motivational outcomes. While recognising that this represents a simplification of the interrelatedness of many factors and their consequences, we hope this aids readers' appreciation of the intervention's context and their understanding of how an intervention delivered at the hospital level may or may not influence health worker behaviour.

### Personal level

#### Altruism, prestige and professionalism

Various reasons account for why health workers chose to become health care workers. Older respondents professed to have been attracted to join healthcare by the altruistic nature of the service (rewards associated with caring for others) with some nurses liking nursing: 'I like nursing because it is a helping profession, just like being a Pastor in a church' [FGD MCH Nurses, H5]. Other health workers joined due to the prestige associated with medical work. The attraction of hospital work might also have been additionally influenced by working with skilled colleagues, especially if working with them resulted in appreciation by patients and/or their relatives.

'Sometimes when the patients become well, they return and give you a chicken kama shukrani kwa kazi mzuri uliyofanya' (as thanks for the good work you did). [FGD MCH Nurses, H5]

Whatever the reason for joining, a strong sense of professional attachment subsequently reinforced by training or organizational/professional ethos was commonly reported among all age groups.

#### Job security

In addition to these, young respondents also stated that they were influenced by the job security offered by health care work (discussed hereafter). It was thought that 'the only problem with working for MSF (Medecin Sans Frontieres, a non-governmental organization) is that one can be sacked any time. With the government, it takes time. They have to find out what went wrong' [Nurse, H3]. Despite appreciating the advantages that government employment provides, some workers took advantage of this situation. As one hospital CEO stated, 'there are people who can't change because they are benefiting from the system. You see that? And there is that element, civil service – nothing can be done to me ... I will get my salary' [Medical Superitendent, H3].

#### Unmet expectations

Perceptions varied between older and younger respondents, the former resigning themselves to working for a future that had increasingly become gloomy. This was attributed to unfulfilled expectations because the conditions of service had deteriorated from the late 1980s through the 1990s when many of them were recruited.

'We just work because we need to, but we are not happy. Even if we retire, utaninginia kwa kaburi kabla ya kupata marupurupu yako (you will teeter by the grave before you get your benefits).' [Small Group Interview of Nurses, H6]

The younger workers in comparison were happy just to have a job, but did not trust the system to look after them in the long term. For example, a few of the young workers accepted the fact that 'salary is a significant de-motivator but I have no problem with it at the moment as I am looking for experience and move on' [CO, H1].

#### Challenged by the demands of clients

Workers sense of fulfilment was challenged by inability to meet the obvious need and high expectations of clients. A medical doctor explained why he found working in his local area difficult:

'You know when you come from the local area na watu wako wajue unado job hapa, masocial zinakuwa mob [and your people know that you are working here, you get many patients (referring to patients coming from his village)]. They come, mafriends, maneighbours na marela (friends, neighbours, and relatives) to get assistance from me ... they report to me kabla ya kuingia hosi (before registering as patients in the hospital).' [Medical Doctor, H2].

### Organizational (hospital) level

#### Physical constraints

Reported constraints affecting health workers' ability to serve patients include shortages of staff, drugs, and non-medical supplies, often in combination with old buildings that resulted in 'staff just work [ing] to clear the queue but not to provide quality work. They do not see the problem of the person' [CO, H7].

System performance is affected in a knock-on sense if there are considerable numbers of workers having multiple roles that they have little time to perform well. This is the case where senior officers working in the hospitals get extra duties at the district headquarters and are not available to carry out their hospital based functions, stretching the abilities of those who work underneath them:

'The pharmacist who runs the hospital is also responsible for the district which has many training functions. This leaves me alone to run the hospital pharmacy.' [Pharmaceutical Technologist, H5].

This has system-wide implications for recent governmental management interventions aimed at improving hospital and worker performance, such as the introduction of the Rapid Results Initiative (RRI). The RRI seeks to introduce systemic changes in the health system. Hospitals develop targets on issues of national importance and agree to meet these in one-hundred days. However, shortages of staff with those remaining having multiple roles has led to questions about such initiatives:

'RRI has been badly affected by the shortage of staff, especially in the running of ARVS due to the high HIV/AIDs rates in the district. Do you know that they [hospital management] have been refusing staff to go on leave in order to meet the targets? The question is that RRI will remain and staff will have to go on leave – so what will happen?' [CO, H7].

#### Relationships between colleagues

Constraints at the workplace could also be attributed to problems with local supervisors who do not appreciate some health workers but instead look for mistakes leading to tension between workers:

'They are not supporting the nurses at all. The doctor comes, he will do the reviews, off. But the nurse is left with that patient. Come to night duty we have almost 60 patients in post-natal with one nurse plus how many beds – eight ... eight ... 16 beds ... 18 beds ...' [Nurse, H6].

Both nurses and doctors reported the CO cadre to have relatively poor inter-professional relations with them, with particular concerns expressed over their performance. However, one senior CO felt differently about the situation, stating that 'My COs feel sandwiched between doctors and nurses. They feel like endangered species as if anything bad happens, it is blamed on them. If everything is okay, they do not seem to appear' [District CO, H3].

#### Lack of fairness

Lack of fairness in ensuring equal access to opportunities, such as training seminars, can be de-motivating:

'At times, in-charges [ward supervisors] get people from their own tribe. There is a lot of ethnicity in the hospital among the supervisory level but not in the lower cadres. The administration also functions along ethnic lines and is not good.' [Nurse, H7].

The perception of fairness must also extend to dealing firmly with indiscipline:

'COs really protect one another – so bad officers go unpunished. If a nurse reports that there is no CO and calls a doctor to see patients, the nurse will be harassed – she is caught in between the two.' [Acting Hospital Matron, H7].

#### Lack of incentives

Even though many issues that cause low motivation cannot be resolved at hospital level, our work reveals that hospital management can work to mitigate low staff motivation. There were some examples of how some simple, local, non-financial incentives might help, such as offering lunch to staff working in critical areas or providing a separate room where hospital staff (and their families) can come for treatment when sick. One doctor felt that:

'They can at least offer tea . look, we chase patients to pay fees. For example, take the issue of filling NHIF forms *[National Health Insurance Fund]*. This is an extra load on us, it is a clerical job. The hospital can earn as much as 200 K (KES 200,000) per month from the forms alone but none of this is ever used to reward or provide incentives to us. So, if they do not give us some of it, it gets lost. You know, the forms pile up and if not claimed within three months, the money is lost.' [Doctor, H2].

On the other hand, careful thought must be paid when considering either changing ways of doing things or withdrawing instituted perks on worker motivation. For example, 'the hospital was providing 10:00 a.m. tea. With the beginning of the new year (2006), the new med sup [medical superintendent] said that there was no money for this facility and it was stopped. People work to generate money but it is not clear what uses the money is put to when generated.' [Nurse, H7].

#### Recognition and appreciation

Recognising and appreciating workers' efforts to do a good job were apparently important influences improving motivation and may have trivial financial implications. However, respondents in some settings argued that although the hospital management was in a position of influence and could improve their motivation to work, they did not take up this role:

'A little effort by the med sup to have, say, an annual process of recognising staff say, Nurse, CO, Doctor, etc would really help staff to realise that the management was watching what they do and would reward good work.' [Senior CO, H1]

That managers did not bother with this aspect of staff management has made many health workers feel unappreciated:

'Like I remember when I was in Siaya, the Medical Superintendent there started this initiative when he was there, so he picked CO of the year, nurse of the year, laboratory staff of the year. The CO was given a wall clock, nurse was given a set of cups, I think it was encouraging – somebody is seeing what you are doing. So somebody, another person will also say if so and so got, why can't I struggle?' [Senior Orthopaedic CO, H1].

#### Communication

A considerable part of good management is good communication between hospital management and its staff. However, most respondents felt there was little communication, and if it took place it was often performed poorly:

'They are the right people, they just need to improve at least communication. Communication is very good to an adult, when you are told wait, you are able to wait ... this one is not possible but if we tried this one, we can try it. Yes, at least there is some communication. But if somebody keeps quiet then you don't know if you are doing the right thing or you are not doing the right thing.' [Nurse, H6]

#### Commitment of managers to improve staff conditions

Despite the preceding, health worker motivation seemed improved in the sites where the hospital director personally took charge and created favourable working conditions to which staff responded positively:

'So then I became a bit committed to my work because people were willing, systems were moving, high bosses have been very supportive, the NGOs [non-governmental organizations] have been coming and they are very supportive and I have found things moving.' [Medical Superintendent, H3].

However, in some settings where the hospital director could have been willing to try and improve work conditions thus staff motivation, the staff were so poorly motivated that they were no longer willing to reciprocate with improved performance. For example:

'The med sup has done much work to improve the hospital. You know, the people here are very difficult. You cannot be soft with them. That is why the med sup is a tough person – that is the only way you can get things done over here.' [CO Intern, H4].

### National context

#### Schemes of service

Salary levels and promotion procedures are outlined in a health worker's scheme of service. In all interviews and across all cadres, both salaries and the way promotions are handled were mentioned to be significant de-motivators. In particular, the lack of promotions was mentioned as a major issue because it affects upward progression and therefore salaries:

'This business of staying for too long in one job group it really de-motivates not just COs in fact all health workers ... it's really de-motivating. It's really, really de-motivating because it's as if you are working, nobody is seeing and nobody is appreciating so you have time and time until you say let me try a greener pasture somewhere.' [Senior Orthopaedic CO, H1].

Even where promotion was possible, there was a clear breakdown of trust between workers and the central bureaucracy:

'Promotion is said to be automatic but this is only on paper. In practice, one has to bribe.' [Hospital Pharmacist, H3]

To some workers, a cadre's scheme of service was a reflection of the way they were recognized and appreciated. For example, the existence of different outcomes from doing similar work with similar levels of risk exposure results in feelings of unfairness:

'There is no risk, uniform, travelling or extraneous allowances yet we work every day and are taxed. For example, a CO's travel allowance is 3 K [KES 3,000] yet doctors get 50 K [KES 50,000] and they come from the same place.' [District CO, H5].

Another example is the provision of the non-practice allowance meant to attract medical officers back into Kenya's public sector that increases their salaries with the proviso that they do not practice privately. The sense of injustice felt by other cadres is compounded by the fact that doctors continue with private practice even though they continue getting the non-practice allowance:

'COs are not considered like doctors ... we are not allowed to practise and are not given a non-practising allowance like doctors. We serve the same government, so we should be given the allowance.' [District CO, H3].

Low salaries were reported to de-motivate staff not just because of unfavourable comparisons with other workers, but because they threatened staffs' ability to meet their daily needs and have a standard of living befitting of their professional status in the community. This further affected their retirement benefits as pensions are pegged on the salary at the point of retirement:

'The new government increased salaries but made the ones of senior staff to be very high and did not touch the salaries of the lower cadres. We have been trying to calm the COs but I feel that they [COs] are not for what we are advising them.' [District CO, H5].

#### Career development

Many COs felt that their cadre was much maligned considering the opportunities available to their colleagues (*i.e*., nurses and doctors) to progress upwards. For example, 'Nurses can start from certificate to PhD. Why not COs?' [CO Intern, H8]. This has been attributed to a poorly functioning scheme of service for COs that has not been reviewed in many years. As such, a senior CO felt that 'there are many hindrances even at the council level. The nurses' scheme has been okayed while the CO one was refused. The question is why are there so many hindrances? It really demoralizes them [COs].' [District CO, H8].

Even where opportunities for self-advancement through training are possible, increased costs of training represent a major barrier. The increased costs are attributed to first, the government reducing or stopping altogether subsidized training for most officers, and second, increases in fees as institutions seek to recoup the lost government subsidies from students.

#### Implications of low motivation

The combination of poor salaries, lack of promotions, and poor access to training opportunities amongst other factors result in low motivation. Poor performance and lack of concern about performance are likely results resulting from the feeling that 'there is nothing to make us feel that we should work' [CO, H8]. In addition, performance is also threatened by burnout resulting from a combination of factors ranging from hospital-related issues, such as heavy workloads and lack of medical supplies to the way staff relate to the community where the hospital is located:

'We see a lot of burnout among staff which has resulted in poor attitudes to patients and work. This has been compounded by poor working conditions and negative attitude from the community.' [Hospital Matron, H5].

Typical reactions include deliberate absenteeism where 'staff just collude with the COs to get sick-offs and then some of them go out to work in private clinics in town' [Hospital Matron, H6]. Another response is lack of timeliness, with some hospitals having introduced attendance registers to ensure that officers came and left on time, although it is difficult at the hospital level to determine whether these have worked:

'They [hospital staff] have started clocking in as a result of the laxity, though, even if they come in on time, it is not known if they are working well or not.' [Hospital Matron, H6].

Other adaptive responses resulting from low motivation and poor remuneration included being 'casual in their approach to work or ... demand [ing] bribes or sell [ing] the drugs given to them by medical representatives' [District CO, H2].

## Discussion

### What are the main findings?

The reports in our work alluding to poor communication, lack of transparency in decision making, an impenetrable and unfair bureaucracy, poor infrastructure, and few resources all resonate with much published work from low-income settings [8,10,11,13]. However, at the hospital level where strong and supportive leadership was present, worker motivation appeared to be higher than in sites that lacked this. This was seen to be critical to improving worker motivation in sites where workers faced significant shortages in equipment, tools and supplies.

This reiterates the important role that hospital management, especially the hospital CEO, has in mediating the effect of de-motivating factors at institutional or national levels. For example, it is posited that the hospital CEO has some leeway to provide local incentives that can improve worker motivation which need not have major financial implications. Examples include identifying and rewarding well-performing health workers. This sends the message that the hospital management is interested in and rewards good performance.

Additionally, good working relationships between cadres also enhance worker motivation. This can be facilitated by the hospital management, for example by holding weekly morbidity and mortality meetings attended by representatives from all cadres where issues affecting health workers' performance can be discussed fairly and decisions made that are followed up. Where inter-cadre relations have been found to be poor, low staff retention, job satisfaction, and inefficiency of health care delivery have been experienced [28], as is the case in Nigeria [29].

However well the hospital management works to create a supportive working environment in the hospital, it is clear that there are issues at system level that affect the motivation, and therefore performance, of health workers. We found examples of Kerr's [30] argument that many systems reward behaviours that they are trying to discourage, a finding similar to those reported from countries such as Mali [8], Ethiopia [11], and Uganda [10]. For example, recognition of worker's efforts has little cost implications yet is not done [8,10,13,31], while staff who shirk their duties or are rude to patients seem to be rewarded by the long period of time it takes to sanction them [14]. On the other hand, if the health system appears to 'favour' a certain cadre through provision of incentives in order to retain them, it is likely that feelings of injustice by other cadres will emerge leading to de-motivation. This in the Kenyan system is apparent between doctors (who have numerous allowances and clear career prospects) and COs who, as substitute physicians, have significantly lower levels of pay and benefits.

It is thought that a major factor creating conditions likely to reduce motivation is the actual implementation of the schemes of service in place [32]. Properly functioning national schemes of service could greatly enhance worker motivation, because every health worker would be treated and remunerated fairly for what they do. In keeping with literature from other countries, inadequate salary and problems with promotion were mentioned by all interviewed health workers as being very de-motivating, being particularly related to retirement benefits [10,11,13,14,31]. In Kenya's health sector, this is perhaps exacerbated by feelings of unfairness. Within the health sector, and as described above, doctors have been receiving a number of allowances aimed at improving their recruitment and retention rates, while COs and other paramedics have not received such financial incentives. In addition, comparisons with other non-health government employees, such as those in the uniformed forces or teachers who also offer essential services but have had their salaries increased, are unfavourable perhaps further contributing to feelings of injustice. While the hospital management cannot directly rectify issues related to delayed promotions or poor salaries, the hospital management can at least act as advocates for their staff. Such actions rely on having good communication channels, often absent, that ensure all are clear on what is possible to help manage health workers expectations of local management.

In theory, there exists in the hospitals studied an annual performance appraisal process, but this appears not to be linked to worker rewards or sanctions. Dieleman and her colleagues [8] found in Mali that appropriate performance management (*i.e*., job descriptions, supervisions, continuous education, and performance appraisal) can positively influence the main motivators of health workers (*i.e*., responsibility, training and recognition, and salary). It is thus vital that initiatives such as the recently introduced public sector performance improvement initiative, of which the rapid results initiative is a part, are not just a paper exercise.

In the setting described, reinforcing a health worker's reasons for becoming a health care worker and attachment to their profession by providing a working environment that supports their work would seem powerfully motivating. In this light, difficulties in the health system that affect the ability to work well undermine a health worker's self-worth and commitment [28], a finding similar to that observed by Kyaddondo and Whyte in Uganda [10]. In our study, sites that were able to support workers' professional identity coupled with continuing professional education (CPE) were found to provide a generally more motivating environment than those without these features.

### Which factors can the planned intervention address and how?

Woodward [1] argues that a hospital must provide an environment where attempts to introduce change will be positively rewarded and that removing cues that make health workers revert to their old behaviour will continue to support change [1,22,23]. Thus, features of sites with environments that could help accept change might include supportive leadership ensuring workers have good access to tools and medical supplies. Other features include a hospital management that creates opportunities for its health workers to access training, use of simple local incentives to positively influence worker motivation and collaboration with civil society, and donors to improve hospital facilities. Few of these characteristics were apparent in the sites we studied.

Instead, a range of problems in all sites were reported, such as sometimes poor teamwork across cadres, significant shortages of resources, inadequate infrastructure and mistrust in the decision-making process particularly with regard to training. These difficulties at the hospital level were compounded by major, national level issues, such as inadequate schemes of service, mistrust, and low salaries. Although the number of hospitals (eight) included in the study is relatively small, we believe that our description of these sites is likely to be representative of a large section of the rural government hospital sector in Kenya.

### Strengthening health workers professionalism

In all eight sites visited, health workers expressed the need to upgrade their skills but lacked the funds to undertake courses that addressed this. The multifaceted intervention being introduced in these sites aims at implementing evidence-based clinical practice guidelines (CPGs) and improving the quality of care being conducted in Kenyan hospitals [16]. The guidelines summarise the available evidence on major diseases and indicate that good care can be provided after relatively brief training with only basic resources [17]. To support the implementation of guidelines, local facilitators from within the hospital are to be provided to encourage the provision of good care, liaise with administrators, and help solve problems related to supplies and equipment [19,20]. The intervention could therefore improve worker's motivation and, when linked to positive feedback, could further encourage good performance [16,17,20]. In this regard, setting clear standards of what is expected, fostering teamwork, and being able to recognise progress towards these standards may be helpful.

### Reinforcing supportive leadership at hospital level

Another major aspect of the intervention aims to improve hospital and health worker motivation and performance through supportive supervision from credible peers linked to feedback on performance and possibly benchmarking with other hospitals [20]. By monitoring how well the hospital has performed in certain preselected and modifiable criteria, shortcomings can be identified and actions taken to improve performance in the hope of introducing a virtuous cycle of improvement [20]. Such effects will depend on the relationships between implementers and hospitals' management, and would benefit from development of the hospital's leadership towards providing as good a working environment as feasible. Ideally, these institutional initiatives would be combined with changes in the national health system context that should include increasing the health workforce and improving resource availability, better remuneration, reliable and transparent implementation of rules, and greater recognition of good service.

## Conclusion

It is clear factors influencing health worker motivation are interlinked, complex, and operate at different levels. While most of those at a national level currently negatively influence health worker motivation in Kenyan district hospitals, it is noteworthy that some improvement in motivation can be attributed to how well a hospital's management organizes and runs the hospital. Workers' financial considerations cannot be gainsaid; however, implementing simple non-financial measures to improve worker motivation may also have some effect. However, interventions that aim to change worker practice simply by offering training are likely to fare poorly unless attention is paid to those factors influencing the motivation of health workers to change and perform well at individual, organizational, and system levels.

## Competing interests

The authors declare that they have no competing interests.

## Authors' contributions

ME conceived the idea for this work and obtained funding to support it. The working approach was developed by PM with support from the other authors. All fieldwork was conducted by PM who was primarily responsible for the analyses and drafting the manuscript with contributions from all authors. All authors contributed to and approved the final manuscript.
